# The crucial role of ethical hospital administration in neurosurgery education

**DOI:** 10.3389/frhs.2022.860266

**Published:** 2022-07-29

**Authors:** Naci Balak, Prabin Shrestha, Kayode Agboola

**Affiliations:** ^1^Department of Neurosurgery, Istanbul Medeniyet University, Göztepe Hospital, Istanbul, Turkey; ^2^Department of Neuroscience, B & B Hospital, Lalitpur, Nepal; ^3^Department of Neurosurgery, Institute of Neurosurgery, A.P. Romodanov, National Academy of Medical Sciences (NAMS) of Ukraine, Kiev, Ukraine

**Keywords:** ethics education, hospital administration, hospital management, neurosurgical residency, organizational ethical behavior, patient safety, professional autonomy, surgical training

## Introduction

Ensuring patient safety is the foremost principle in surgery. Neurosurgery, which is a high-technology dependent, high cost and high-risk surgical specialty, is one of the most demanding branches of medicine (1–9). Professionalism and patient safety cannot be fully ensured by providing only technical skills during neurosurgical residency training (1, 10). Continuous ethical education is also essential for competent medical practice. Therefore, formal ethics curricula for the professional training of residents have been introduced in several countries (10). However, moral practices are required not only from individuals but also from systems and institutions as a whole (10–13).

All decisions and practices within institutions directly affect ethical healthcare. The climate of change within which healthcare is provided, such as population growth, aging, increased demand for addressing chronic conditions, shortage of resources and the rapid growth of technological advancements, which includes diffusion of innovation and digital health revolution, can affect healthcare and its ethics (13). For instance, the rapid growth of technology can bring about challenges for administrators in the areas of finance, staffing and patient demands (13). The crucial effects of administrative decisions came to light very clearly during the COVID-19 pandemic (14–20). The inadequacy of available medical resources, such as the scarcity of intensive care beds, necessitated institutional decisions which may have adversely affected the provision of basic medical needs for neurosurgery patients.

Hospital managers are expected to provide accountable and optimal distribution of resources and to avoid conflict of interests between themselves and the needs of society and of the individual patient (21). Furthermore, the creation of an ethical climate in hospitals is essential for the fostering of an educational environment open to ethical discussions. Unfortunately, resident physicians in a strict hierarchical system where they do only what they are told are at risk of failing to grasp ethical issues. Government policies and hospital management can conflict with patient preferences and/or resource constraints and ultimately undermine traditional medical practice and education (1). To avoid creating global disparities in neurosurgery, trainees must have to understand the ethical dimensions of power imbalances, priority settings, the role of funding mechanisms in driving clinical care and research (22).

## Ethical structure in hospital administration

The creation of the ethical climate is necessary for the establishment of a robust resident training. Two main factors underpin ethics in hospital administration. The first of these is the obligation to ensure the autonomy, priority and safety of the patient, and the second is the personal ethics of the healthcare manager as the moral representative and leader of the hospital (3, 11, 13, 23, 24).

Virtually all administrative decisions that arise in managing health services have ethical dimensions (11–13). These ethical issues are qualitatively distinct from those encountered in the business world. In business ethics, profitability is the only goal in the framework of established law (2). Furthermore, the concept of respect for individuals—which emphasizes autonomy, fidelity and confidentiality—is essentially absent in business world. Moreover, the principle of justice is found only at the periphery of business ethics. It has been argued that even guidelines such as “the Hospital Financial Management Association Code of Ethics” and “the Guidelines on Ethical Conduct and Relationships for Health Care Institutions” adopted by the American Hospital Association fail to prevent hospital administrations from giving priority to economic goals, either explicitly or implicitly (25–27).

## Contemporary hospital administration

Increasingly, healthcare systems are managed according to the principles of new public management systems (1). However, this approach leads to a perceived loss of professional autonomy for physicians and trainees (1, 3). Physicians are required to be autonomous and make independent decisions. The tension between physicians' demands for the individual patient always to come first, due to their professional ethical obligations, and the requirements of the health institution, which usually forces attention to the needs of the community rather than to a single individual, is an ever-present source of conflict in today's healthcare organizations (28, 29). Furthermore, technological advances such as the digitization of the healthcare industry, the use of big data and deep learning, artificial intelligence applications, robotic skull base surgery, virtual reality application, focused ultrasound, and new application areas of deep brain stimulation have undoubtedly led to the transition to more complex treatments in neurosurgery (3, 5, 8, 30–35). Parallel to this, complexity in hospital administration and imbursement structures have escalated.

Meanwhile, performance management, which is one of the most important tools of current public management methods, carries the risks of causing problems because it requires time consuming and burdensome bureaucracy with measurement parameters which may not always be appropriate. No matter how optimized the measured parameter is, the use of surrogate parameters can be impractical or even destructive in the evaluation of the original object of interest. For example, the length of hospital stay of patients is a common variable (36). With this variant, a short stay is considered favorable. However, if a surgeon's daily mortality is 100 percent, the length of stay will be very short and the surgeon will be found to be paradoxically efficient (1). As another example, using “complications” as a measurement parameter may lead surgeons to select simple, less risky cases to avoid this negative efficacy parameter, and not only may patients with complex problems remain untreated but also resident training may be adversely affect (1). Thus, new public management systems, in which proxy parameters are used to measure efficiency, seem to have increased “efficiency” but negatively affected the focus on education and scientific research (1).

## Qualities of administrators: morality, power, and leadership

Administrators tasked with keeping costs under control in order to organize and sustain the provision of health services will seek to reduce costs and maximize efficiency (3). Given these challenges, pragmatism may make the application of ethics seem less important. For example, it might be expedient to ignore the mission, vision and values of an organization when using the argument that costs are the most important factor for survival of the healthcare system.

Although healthcare administrators may need to take additional issues into consideration, they must nevertheless provide an environment where patients receive both appropriate and compassionate care. They must respond to the business needs of healthcare while respecting the patient, staff members, organization and society. Administrators also represent the political owners of power. Unfortunately, there is evidence that power has the potential to corrupt (37–39). Power may often lead people to place their own interests above the needs and goals of others. Power liberates individuals to focus inward, leading them to place greater weight on their own aims and interests. Power also appears to cause individuals to “objectify” others, to see them as tools and to see relationships as peripheral in nature. This may lead to administrators making self-interested decisions when faced with ethical dilemmas.

The regrettable reality is that leadership power is frequently abused. According to research, administrators are accountable for 60% of workplace misconduct (40). In workplace settings there are common ethical concerns including immoral leadership, a toxic workplace culture, unachievable and conflicting goals, the misuse of tools and technology and employee discrimination and harassment based on certain parameters such as color, ethnicity, gender, handicap, or age (40–42).

It will be very difficult to attract the best and brightest medical students for residency training in a teaching hospital without an ethical climate. Educational environments where gender, ethnicity, religion, culture, sexual orientation or identity, socioeconomic strata or any other individual identifying characteristics are the cause of mobbing will harm the future of our profession (43). The underrepresentation of neurosurgery in undergraduate teaching and training further increases the importance of creating an ethical climate (44). In particular, the fact that female physicians are not attracted to neurosurgical residency training at a rate similar to their entrance to medical faculties may worsen this situation (45, 46).

The moral framework of managers is their personal ethics, which impact their communications with patients, healthcare personnel, healthcare institutions, insurance companies, governments, and society in general (3). However, it would be wrong to explain the behavior of managers only by their personalities, because the power of social influence cannot be underestimated. So much so that even small changes in the social environment can overcome differences in people's personalities. In other words, how managers perceive, comprehend and interpret the world around them is also important (1).

## Ethical behaviors and practices

From an ethics standpoint, the first step to the application of ethics in decision-making is to understand its definitions, theories and principles (24). The general terms of ethics are relatively well-known. Unfortunately, the problem is that the details are not known. Healthcare administrators must not only have a basic knowledge in ethics—which consists of autonomy, beneficence, non-maleficence, justice, dignity, and honesty—but also be able to apply ethics at a more profound level through appropriate behaviors that maintain both personal and organizational integrity.

Martin Buber was a major theorist who helped to lay the groundwork for health-care ethics (13). Buber centered on the idea of relationships. The least effective human relationship is the “I-I” relationship. In this relationship, the needs of others do not exist, nor does the accountability of moral conduct toward them. The next level is the “I-IT” relationship. Because a person is seen as an “it”, they can be used as a tool for personal benefit or the benefit of one's organization. An example of an the “I-IT” relationship occurs when administrators use such terms as “my people” or “my workers”. Another example is reference to a patient as “room number” or “diagnosis” instead of by her or his name. The third category of relationships is the “US-THEM” relationship. In this category, people are grouped as “us” and “not us”. People who are in the “us” group believe themselves to be superior and avoid dialogue with those in the “not us” group. It is therefore easier to attribute negative events or actions to those who are “not us”. The fourth category of relationships is the “I-YOU” relationship. Only in this category are individuals recognized as having value, unique abilities, gifts, and thoughts. These distinctions are not only acknowledged, but they are also accepted and valued. Unfortunately, administrators may rely on the directing function of management rather than engaging in dialogue with their staff regarding the adaptation process for responding to change.

Hospital managers begin their careers as adults who have an implicit personal ethic. In developing that ethic, managers are affected by numerous influences beyond their own introspection, including family and friends, religious principles and teachings, secular education and the law. While one's basic worldview is tending to remain rather constant, one's personal morals can be enhanced by experience, maturation, and technological advances and education. Administrators can develop ethical decisions through a foundation in ethics theories and principles that can be applied to situations in hospital. They can also engage in dialogue with clinical staff for insight into the practice of ethics.

Hospital administrators should bring together all stakeholders with the ultimate goal of continuously improving healthcare delivery, training, and scientific research (47). This includes a broader scope for public engagement in allocation of research funding and approval of research projects. Hospital administrators, who already play a relevant decisional role, should pay a renewed attention to the issues of equality and diversity in neurosurgery. Thus, they could positively address the unmet needs of unbalanced academic systems where the rank achieved can still differ by gender despite identical performance metrics (48, 49).

Administrators must realize that universal truth in moral human societies does not differ, and it does not depend on the moral norms of the society in which it is practiced (24). A proposition is either right or wrong, but it cannot be both right and wrong. Snap decisions may lead to poor choices. Instead of “right now” decisions, “right way” decisions must be pursued. Few substantial ethical changes come easily without time and devotion. Healthcare administrators should be encouraged to speak up about the ethics concerns related to policies or practices. Concerns can be voiced through attending meetings and asking questions. Patience, courage and persistence are also a part of the formula for addressing ethics in an era of great change.

Robbins suggested that ethics committees be included in the decision-making process for addressing change in healthcare organizations (50). Having ethics resources—such as ethics committees, a well-articulated vision statement and support for practicing ethics—enhances the ability to make changes while respecting ethics practice.

## Conclusion

Moral practices are required not only from individuals, but also from healthcare institutions as a whole and continuing ethical education of trainees is necessary for adequate medical practice.

Professional experience, values-based ethics and trust underpin improved healthcare management. Greater improvements and changes can be made if independent ethics committees are included in administrative decision-making processes.

## Author contributions

NB: creation and design of the study, literature search, data collection and analysis, writing of the manuscript, and revision of the manuscript and final approval. PS and KA: literature search, data collection and analysis, writing of the manuscript, and revision of the manuscript and final approval.

## Conflict of interest

The authors declare that the research was conducted in the absence of any commercial or financial relationships that could be construed as a potential conflict of interest.

## Publisher's note

All claims expressed in this article are solely those of the authors and do not necessarily represent those of their affiliated organizations, or those of the publisher, the editors and the reviewers. Any product that may be evaluated in this article, or claim that may be made by its manufacturer, is not guaranteed or endorsed by the publisher.
